# An Agent-Based Model for Simulating Flood Governance and Community Resilience

**DOI:** 10.1016/j.mex.2026.103820

**Published:** 2026-02-13

**Authors:** Anqi Zhu, Wenhan Feng, Huan Zheng, Yingxin Huang, Xin He, Chengying Zhou, Liang Emlyn Yang

**Affiliations:** a Department of Geography, Ludwig Maximilian University of Munich (LMU), Munich, Germany; b School of Humanities and Social Science, The Chinese University of Hong Kong, Shenzhen, China; c Harmony Community Foundation, Guangzhou, China; d Conservatoire National des Arts et Métiers, Paris, France; e Guangzhou University, Guangzhou, China

**Keywords:** Community resilience, Flood resilience, Governance network, Emergency response, Post-disaster recovery, Social simulation, China

## Abstract

Agent-based modeling (ABM) is a unique tool for understanding social mechanisms and emergent phenomena. The paper presents an empirically grounded agent-based model that simulates how stakeholders embedded in flood governance networks facilitate community loss-sharing and post-flood recovery. The model is designed and calibrated using extensive empirical data from communities in Guangzhou, China. Modeled agents include multi-level government agencies, NGOs, private sector entities, and local clans, among others. The model integrates core processes (rainfall and flood impacts, network-based loss sharing and recovery, and the implementation of resilience measures) with modules for trust evolution and resource constraints. The purpose of this model is to evaluate the effects of different network structures, inter-stakeholder trust, and the diffusion of flood resilience measures on community flood resilience, and to advance the understanding of how resilience emerges as a macro-level attribute from micro-level interactions. Innovations are twofold: First, it moves beyond static analysis to simulate the dynamic, network-based collaborative processes among diverse institutional stakeholders; Second, it implements a process-based framework to measure community robustness and adaptivity, using these metrics to evaluate overall community resilience to floods. Key parameters, derived from literature and empirical research, were validated and tested via sensitivity analysis. The model serves as an accessible tool for researchers and practitioners interested in stakeholder collaborations in community-level climate governance and identifying optimal intervention strategies.

• The model is described using the ODD protocol.

• Validation, sensitivity analysis, and the number of minimum simulation runs are explained.

• Complete NetLogo code and a brief user guide are provided.

## Graphical abstract

**Figure:**
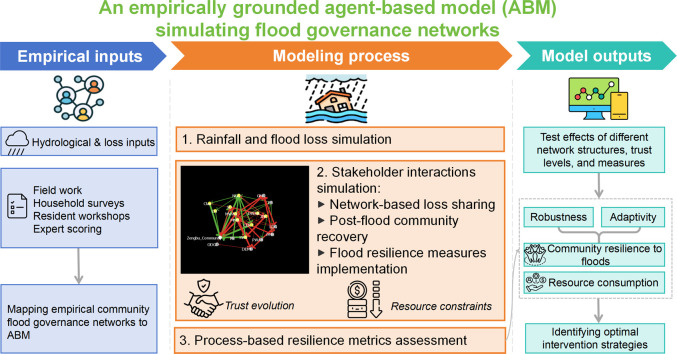

Specifications table

**Table:** 

**Subject area**	Environmental Science
**More specific subject area**	Social simulation
**Name of your method**	Governance network agent-based model (ABM)
**Name and reference of original method**	N/A
**Resource availability**	NetLogo software is available from: https://ccl.northwestern.edu/netlogo/ NetLogo code is openly shared and accessible at: https://github.com/AnqiZhu31/GovernanceNetwork-ABM

## Background

Global climate change presents an escalating threat to human society, with more frequent and severe weather events causing transboundary disruption to infrastructure, food security, public health, and economic growth [1]. In this context, resilience has become a central concept in climate change adaptation and disaster risk reduction across research and practice [[2], [3], [4], [5]]. While resilience has been framed by scholars and practitioners with different emphases, at its core resilience refers to the capacity and process of interconnected social, economic and ecological systems to resist, absorb, learn from, and adapt to shocks or stresses, to maintain essential functions, as well as transform to more desirable configurations [[6], [7], [8], [9], [10], [11]]. We focus specifically on community resilience. From a systems perspective, community resilience is shaped by diverse influences in multi-level, nested relationships, characterized by complexity (e.g., learning, self-organization, feedback, nonlinearity, interdependence, and cross-scale linkages) [7,12]. Assessing resilience remains challenging [13]. One reason is that resilience becomes observable only in the presence of shocks and disturbances [14,15]. Moreover, resilience outcomes emerge from complex interactions between human factors and environmental conditions which are inherently difficult to quantify with large uncertainties [16].

Governance and institutions are critical determinants of resilience [14,17,8]. Governance networks, defined as stable patterns of social relations between stakeholders who center around complex policy challenges, have been increasingly recognized for their potential to enhance coordination and resource sharing across levels in climate adaptation and building resilience [[18], [19], [20], [21], [22], [23]]. Thus, it is essential to unpack how governance networks operate and to examine how network settings shape resilience outcomes, particularly in rapidly urbanizing areas such as China’s Pearl River Delta (PRD) [24].

Agent-based modeling (ABM) offers a powerful methodological approach for capturing how the interdependent behaviors of heterogeneous agents give rise to complex system dynamics, non-linear processes, and emergent phenomena [[25], [26], [27], [28]]. Existing ABM studies have demonstrated the advantages of ABM in incorporating human behavior into environmental change simulations, covering topics such as migration, community infrastructures, evacuation behavior, agricultural adaptation, and shelter planning [[29], [30], [31], [32], [33], [34], [35], [36]]. Beyond behavioral modeling, notable methodological advancements have emerged in coastal structural resilience assessment and property damage estimation [37,[38], [39], [40]]. Yet limited attention has been paid to the grassroots governance practices and institutional interactions [41]. This paper fills this gap by developing an empirically grounded ABM that simulates dynamic interactions among institutional stakeholders involved in community-level flood governance.

The model has two primary objectives. First, it seeks to understand resilience as a system-level emergence from micro-level interactions among heterogeneous stakeholders. Second, it enables comparative analysis of how different governance interventions (variations in network structure, inter-stakeholder trust, and the diffusion of flood resilience measures throughout the network) affect community resilience to floods. This model is designed for exploring mechanisms and comparing intervention scenarios, not for precise flood damage assessment. It provides an experimental environment where users can modify governance structures, trust dynamics, and measures to evaluate their relative resilience outcomes and resource usage.

Key innovations of this model are twofold. First, the model moves beyond static analysis to simulate and evaluate the dynamic, network-based interactions and collaborative processes between diverse stakeholders (e.g., multi-level government agencies, NGOs, the private sector, and local clans) and to further provide evidence on the impacts of governance interventions aimed at mitigating and managing external risks [42]. Second, the model operationalizes resilience by implementing process-based metrics across two dimensions: robustness and adaptivity, based on the social resilience framework proposed by Schweitzer et al. [15]. This approach allows us to observe and measure resilience as a dynamic system performance rather than a static collection of proxy indicators. As demonstrated by Feng et al. [43], this process-oriented measurement with ABM enables the continuous visualization of system trajectories as they absorb shocks and gradually recover, thereby revealing not only the outcome, but also pathways and potential thresholds. Beyond these short-term metrics, the model also reflects the structural transformations for long-term adaptation, as evolved trust and community capacities create a new, stronger baseline for future change.

This article serves as the first open, citable source of the complete ABM design, code, and validation. An empirical application of the model is presented in Zhu et al. [44]. The model offers a replicable and adaptable framework for researchers and practitioners interested in governance networks and community resilience. While the current calibration focuses on flood response in urban areas in South China, the underlying methodological framework is structurally transferable. Fundamentally, the model simulates how dynamic and time-varying interactions among agents that occupy different positions in the network and make decisions with bounded rationality, influence the cascading transmission of information and the activation of resources, ultimately shaping system dynamics under uncertainty. Readers can use the model as a process-based framework for measuring resilience and as a modular ABM codebase to test interventions and counterfactual scenarios. With local environmental and stakeholder data and calibrated parameters, the core module can be adapted to other institutional settings and extended beyond flood risk.

## Method details

The model details are described using the ODD (Overview, Design concepts, Details) protocol for agent-based models [45], as updated by Grimm et al. [46].

**ODD Protocol**

## Purpose and patterns

### Purpose

The purpose of this model is to investigate and illustrate how governance networks influence community resilience to rainfall-induced flooding. The model is used to investigate three key factors: (1) changes in governance network structures, (2) trust levels between stakeholders, and (3) implementation of flood resilience measures. The model focuses specifically on stakeholder dynamics during and after flood events, rather than attempting to simulate the entire community system or generate precise flood loss predictions.

### Pattern

Our model is designed to reproduce the following patterns observed in the communities in Guangzhou, Pearl River Delta, China.

Pattern 1: Community resistance and recovery process

This pattern captures the relationship between flood intensity and community response trajectories. First, the impacts of flood intensity on communities. Communities facing low-intensity floods maintain higher functionality and recover rapidly. Major floods bring more severe damage, leading to a slower recovery process and prolonged restoration period. Second, we use community functionality (initialized at 100) to represent the extent of functional loss during a flood and its subsequent recovery over time. This functionality typically follows a characteristic temporal trajectory: it declines upon exposure to rainfall-induced flood shocks and gradually recovers thereafter, which is similar to the recovery curve of functionality modeled by Fahad et al. [37]. The model should capture this dynamic by illustrating how recovery trajectories vary nonlinearly with flood intensity.

Pattern 2: Influence of trust levels on community resilience

This pattern examines how trust shapes stakeholder collaboration during flood events. High trust among government agencies, NGOs (non-governmental organizations), and community organizations facilitates rapid resource mobilization and efficient coordination. Conversely, low trust leads to coordination failures, delayed responses, and resource allocation inefficiencies [47,48]. Meanwhile, successful interactions will increase the trust between stakeholders [49]. However, as Sutcliffe & Wang [50] note, the relationship between interaction frequencies and trust is logarithmic rather than linear. This implies that successful interactions yield significant gains when trust levels are low, but these gains diminish as trust levels rise. The model should reflect these trust-dependent outcomes by generating different community functionality curves.

Pattern 3: Effectiveness of flood resilience measures

This pattern examines how different flood resilience measures affect community resistance capacity and recovery capacity. Physical measures such as levees and drainage infrastructure primarily enhance resistance capacity by reducing initial flood damage, while social measures including early warning systems and emergency response predominantly facilitate recovery through improved information dissemination and social mobilization [[51], [52], [53], [54]]. The model should reflect these effects by showing improved resistance and recovery metrics when appropriate measures are implemented. Model validation will assess its capacity to reproduce these empirical patterns across varied flood scenarios, thereby demonstrating how governance network structures, trust dynamics, and intervention strategies interact to shape community resilience outcomes.

## Entities, State Variables, and Scales

### Entities

The model includes the following entities: agents representing the community and stakeholders, links between stakeholders in the governance network, and the environment, which represents external rainfall and flood conditions. We define the model boundaries based on the functional flood governance network operative at the community level. While the model centers on local government, public, and market actors, it also incorporates provincial-level agencies. As identified by residents and stakeholders, these higher-level government actors remain indispensable to local governance given China’s top-down accountability system [55]. The selection of the nodes (1 community agent and 18 stakeholder agents) and their links is empirically grounded. It derives from extensive fieldwork and two resident workshops, ensuring the model reflects the key actors and the operative governance network observed in the field. For empirical details, please refer to Zhu et al [44].

The *Community agent* represents the primary unit of analysis in the model, capturing its ability to resist, recover, and interact with stakeholders in the governance network during and after flood events. It is modeled as an aggregate unit representing the collective households that directly bear flood losses. This aggregation places all agents at a commensurate level of analysis, treating the community as an entity comparable to other organizational stakeholders.

*Stakeholder agents* represent 18 diverse actors essential to flood governance, including government departments at multiple administrative levels (provincial, municipal, district, and neighborhood), NGOs, representative private sector entities, the community emergency response team, and academic and research institutions.

*Links* represent relationships and interactions between stakeholders, categorized as either those involving government institutions or non-government actors. The effectiveness and trust levels of these links directly influence the extent to which stakeholders’ responses can contribute to community loss sharing and recovery*.*

The *environment* represents external rainfall and flood conditions at the system level, including key factors such as rainfall intensity, flood loss ratios, and flood duration.

### State Variables

See Table 1.

**Table 1:** Entities and state variables.

Entity type	State variable	Description	Variable types and range
Community	functionality	Represents the community’s functionality level	Float, [0, 100]
	resistance capacity	The community’s capacity to resist flood damage, influenced by flood resilience measures	Float, [0, 1]
	recovery capacity	The community’s capacity to recover post-flood functionality, influenced by flood resilience measures	Float, [0, 1]
	robustness	A metric measuring how well a community maintains functionality during a flood event with the assistance of connected stakeholders	Float, [0, 1]
	adaptivity	A metric reflecting how well a community recovers and adapts to changing environments, combining recovery rapidity and governance network activity	Float, [0, 1]
	resilience	The community’s capacity to resist, absorb, learn from, and adapt to stresses or disruptions, calculated based on robustness and adaptivity	Float, [0, 1]
Stakeholders	type	Government actor or non-government actor	Categorical, government/non-government
	response-probability	Probability of a stakeholder responding to community requests	Float, [0.2, 1]
	response-duration	Duration (in ticks) for which a stakeholder remains active once a response begins	Integer, [1, 5]
	is-active?	Whether a stakeholder has responded and currently stays active	Boolean, True/False
	response-count	The number of times a stakeholder has responded	Integer, ≥ 0
	resource	Remaining resources a stakeholder has available for responses	Integer, ≥ 0
Links	type	Link type indicating whether it originates from a government agency (government-directed) or non-government actors (non-government-directed)	Categorical, government-directed/non-government-directed
	effectiveness	Residents’ perceptions of how effective a specific link with stakeholders can help the community in flood risk reduction, emergency response and post-flood recovery	Float, (0, 1]
	trust	Trust level between stakeholders	Float, [0, 1]
Environment	flood-intensity	Intensity of flooding, categorized by return periods	Categorical, 10-year/50-year/100-year
	flood-duration	Duration of the flood event in hours	Integer, [4, 24]
	disaster-phase	Current phase of the disaster process	Categorical, pre-disaster/during-disaster/post-disaster
	measures-activated?	Activation status of flood resilience measures	Boolean, True/False

### Spatial and Temporal Scales

(1) Spatial scales

**Fig. 1:**
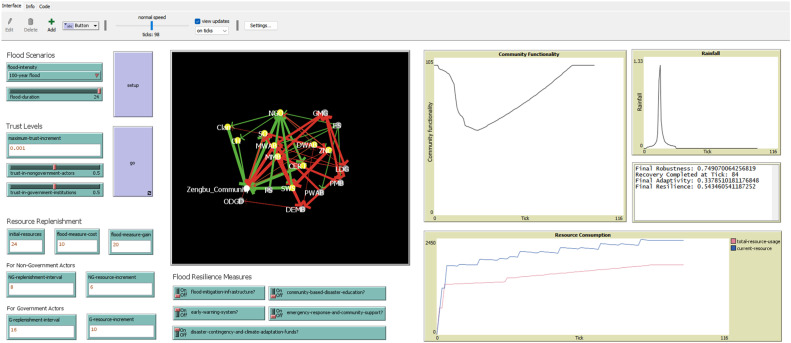
An example snapshot of the model, as visualized in NetLogo.

The model uses a social network structure rather than geographic space, as shown in Fig. 1. Stakeholders (communities, government agencies, NGOs, and private organizations) form the network nodes, connected by links that represent their interactions, resource flows, and information sharing during floods. By focusing on relationships rather than physical locations, the model can be applied to different governance contexts without being tied to specific geographic settings.

(2) Temporal scales

Time is represented through discrete steps. Each tick represents one hour. In accordance with official guidelines from the Guangdong Climate Center [56], rainfall duration is characterized by intervals ranging from 240 to 1440 minutes. Consequently, the duration of flooding caused by these heavy rainfall events is represented in the model as lasting 4 to 24 ticks (4 to 24 hours).

## Process Overview and Scheduling

### Process

The ABM simulates flood governance networks and community resilience through discrete time steps. The model captures the complete flood cycle. The simulation proceeds through three phases.

(1) The pre-disaster phase initializes the governance network with flood parameters and baseline trust levels.
(2) The during-disaster phase captures rainfall, flood impacts, and stakeholder coordination of loss-sharing through network interactions.
(3) The post-disaster phase focuses on community recovery through continued interaction with stakeholders.

At each time step, the model updates environmental conditions, community functionality, stakeholder resources, and trust levels. The flood resilience measures can enhance the community resistance and recovery capacity. Two resilience metrics are computed: 1) Robustness is assessed at the end of flood to measure functionality preservation. 2) Adaptivity is evaluated after recovery to reflect recovery speed and the active level of the governance network.

### Schedule

**Fig. 2:**
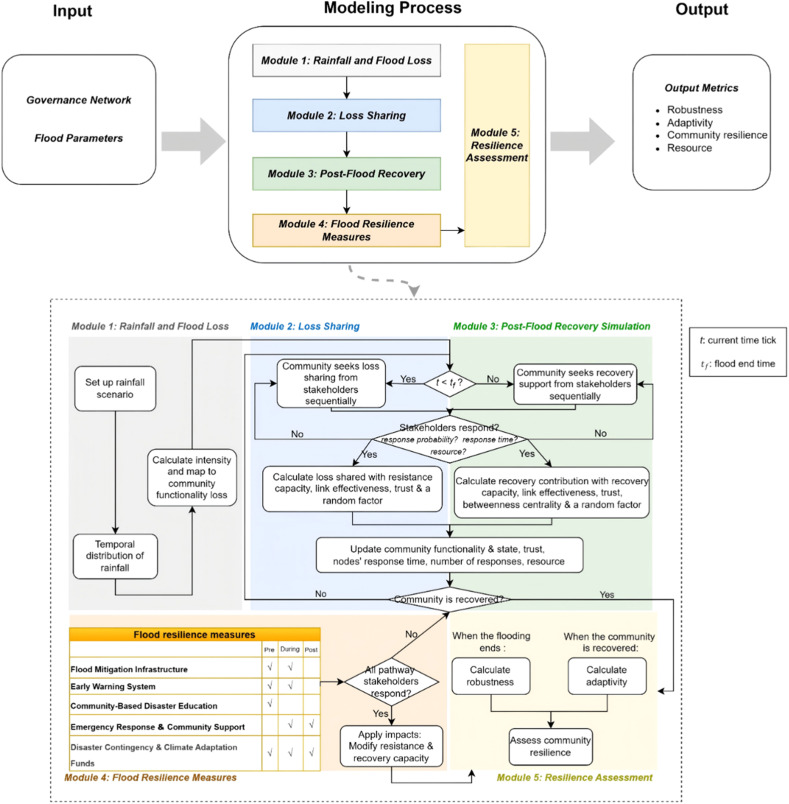
Modeling procedures.

Fig. 2 shows the modeling schedule. Following data input on the governance network and flood parameters, the model operates through five key modules.

First, the simulation of rainfall and flood losses begins during the pre-disaster phase. Rainfall is generated using Guangzhou’s official rainstorm intensity formula and the Chicago Design Storm, simulating return periods of 10, 50 and 100 years, with durations ranging from 4 to 24 ticks (hours).

Second, loss sharing is executed through network-based response mechanisms. Communities request assistance from networked stakeholders in sequence. Stakeholders’ contributions are determined by trust levels, link effectiveness, community resistance capacity, and stochasticity. After each tick, the model updates community functionality, trust, resources and other variables.

Third, the recovery process initiates after flood cessation. Stakeholders support community recovery based on their betweenness centrality, link effectiveness, trust levels, community recovery capacity, and stochasticity. This process continues until the community achieves full functionality restoration. It also updates trust and other variables similar to the loss sharing process.

Fourth, flood resilience measures are conditionally activated based on specific disaster phase timing. These measures affect the community’s capacity to resist and recover from floods. Finally, the model assesses community resilience from two dimensions: robustness and adaptivity.

BehaviorSpace is used to assess and compare the impact of governance interventions and institutional settings on community resilience. Users can run batch simulations that vary with key parameters, including different governance network structures, initial inter-stakeholder trust levels, and the activation status of flood resilience measures. This process allows us to compare how different combinations of interventions quantitatively affect community resilience.

## Design Concepts

### Basic Principles

The primary objective of this model is to simulate how stakeholders within governance networks support communities in reducing flood impacts and facilitating recovery during flood events. At the agent level, stakeholders optimize utility by minimizing flood losses and maximizing recovery speed through collaborations, constrained by available resources and capacity. At the system level, network structures, trust relationships, and resilience measures determine overall system robustness and adaptivity. The model examines how governance structures facilitate or constrain collective flood response.

### Emergence

Community resilience emerges from local, resource-constrained interactions among governance actors. Stakeholder nodes activate in a nonlinear sequence, first mobilizing support through their closest connections and then expanding outward, continuing to assist until their resources are depleted. These activation dynamics produce a nonlinear recovery trajectory, as community functionality shifts from immediate loss toward gradual restoration. The model further incorporates feedback loops: each successful collaboration increases trust, which in turn enhances the effectiveness of subsequent interventions. And early interactions generate relatively larger trust increment, while subsequent gains become progressively smaller.

### Adaptation

The model incorporates three adaptation behaviors. First, stakeholders demonstrate phase-specific responses by shifting from loss-sharing assistance during floods to recovery support post-flood. Second, agents dynamically update trust levels based on successful collaborations, with increased trust immediately affecting subsequent engagement effectiveness. Third, agents decide whether to respond based on available resources, with agents unable to respond to community requests when resources fall to zero.

### Objectives

The model aims to minimize flood impacts and maximize community resilience through coordinated governance under resource constraints. Agents pursue different objectives across flood stages. During the flood, they prioritize loss sharing for the community. After the flood, the focus shifts to community recovery.

### Learning

The model does not include individual agent learning.

### Prediction

Prediction is not included in the model.

### Sensing

Stakeholders sense community needs and recovery status, ensuring that assistance continues until a community has fully restored its pre-flood functionality. Network nodes perceive trust levels of directly connected neighbors. The spatial range of sensing is limited to adjacent nodes, meaning agents rely on localized information when making decisions.

### Interaction

Direct interactions occur when communities send loss-sharing and recovery requests to nearest linked stakeholders. These requests propagate step by step through the network. A successful response from a node enables loss-sharing or recovery for the community. Additionally, each successful interaction reinforces trust. Resource-depleted stakeholders cannot respond.

Indirect interactions arise from resilience-enhancing measures that are introduced by a given node and disseminated through empirically derived network pathways. They influence flood resistance and post-disaster recovery capacities, thus shaping the final resilience outcome.

### Stochasticity

Stochasticity in two processes yields non-deterministic outcomes that reflect variability in flood governance.

(1) *Probabilistic response behavior*. Stakeholder nodes respond to community requests based on probabilities. This reflects differences in capacity, willingness, and situational constraints, preventing uniform or overly deterministic responses. The same logic applies to the diffusion of resilience measures, though their transmission pathways are pre-defined.
(2) *Contribution effectiveness*. When responses occur successfully, a stochastic factor enters the calculation of link effectiveness, mimicking variability in real-world aid distribution.

### Collectives

Collectives are not considered in the model.

### Observation

The model observes community functionality, robustness, adaptivity, resilience and resource consumption across flood phases.

## Initialization

See Table 2.

**Table 2:** Initialization of the model.

Aspect	Details
Community node	pre-flood functionality: set to 100 resistance capacity and recovery capacity: both set to 0.5 recovery status: set to false
Stakeholder nodes	type: government actor or non-government actor response probability and response duration: input data activation status: set to false response number: set to 0 set initial resource and resource replenishment parameters
Links	type: government-directed or non-government-directed link effectiveness: input data
Trust	type: trust in government actors and in non-government actors baseline trust: 0.5
Flood scenario	set flood intensity, flood onset, flood duration flood loss ratio: input data

## Input Data

See Table 3.

**Table 3:** Input data.

Data type	Description	File format	Source
Network data	Describes the governance network, listing all stakeholder nodes and the links between them. Each link includes its direction, link type, and link effectiveness.	Empirical data transferred to CSV file	Collected through household survey (Nov 2022–Feb 2023), fieldwork and 29 in-depth household interviews (Feb 2023–Feb 2024), two resident-centered participatory workshops (Oct 2024), and expert scoring (Oct 2024) involving four experts in flood management and community development.
Node data	Contains attributes for each node, including response probability, response duration and node type.		
Rainfall and flood loss curves	Provides formulas and parameters for rainfall intensity with return periods of 10, 50 and 100 years, as well as flood distribution patterns and related flood loss ratio. These establish the relationship between rainfall intensity, duration, and direct flood losses in Guangzhou.	Embedded in model code	Rainfall data and storm parameters are based on official guidelines from Guangdong Climate Center [56]. Flood loss assessments are based on empirical studies in Guangdong [57].
Flood resilience measure pathways	Specifies pre-defined pathways for implementing flood resilience measures across different disaster phases.		Developed from fieldwork and expert scoring. Further details are provided in Zhu et al [44].

## Submodels

### Rainfall and Flood Loss Module

This module simulates precipitation and its corresponding losses in our study community in the central urban area of Guangzhou. Key parameters are examined in the sensitivity analysis.

(1) Rainstorm intensity calculation

The rainstorm intensity calculations are based on the Technical Report on the Compilation of Rainstorm Intensity Formulas and Design Storm Patterns for Guangzhou, developed by the Guangzhou Water Authority, Meteorological Bureau, and Guangdong Climate Center [56]. This report synthesizes over 40 years of meteorological data to derive intensity-duration-frequency (IDF) relationships for the central urban area. The model integrates IDF formulas corresponding to three return periods (10-year, 50-year and 100-year floods) to determine rainfall intensity over time.

The design rainfall intensity formulas, expressed in L/(s·hm²), are:

(1) $$q_{10}=\frac{16971.541}{{\left(t_{m}+34.941\right)}^{0.916}}$$

(2) $$q_{50}=\frac{24556.014}{{\left(t_{m}+46.25\right)}^{0.913}}$$

(3) $$q_{100}=\frac{27212.984}{{\left(t_{m}+49.226\right)}^{0.912}}$$

where q represents the design rainfall intensity in L/(s·hm²); $t_{m}$ represents the rainfall duration in minutes in the formula. To integrate this formula with the model’s hourly time step, the input duration tick (in hours) is multiplied by 60 to convert it into minutes.

(2) Temporal distribution of rainfall

To simulate the temporal variation of rainfall intensity, the Chicago Design Storm method is employed, generating time-dependent rainfall intensity curves. This approach allows for the introduction of a peak rainfall intensity at a specific time $\;t_{p}$, calculated as:

(4) $$t_{p}=T_{f}\times r$$

where $T_{f}$ is the duration of the rainfall; r=0.35 represents the fraction of the rainfall duration at which peak intensity occurs. Substituting $t_{p}$ into the storm intensity formula yields the peak rainfall intensity $q_{max}$.

(3) Standardizing rainfall intensity for loss assessment

To ensure compatibility with the flood damage function, rainfall intensity values are normalized within a standardized range of 1 to 5. The standardization formula is:

(5) $$I_{std,t}=\frac{q_{t}}{q_{max}}\times \;5$$

where $q_{t}$ is the instantaneous rainfall intensity at tick *t*; $q_{max}$ is the peak rainfall intensity.

This standardization allows for direct comparison with damage curves used for flood-induced loss estimation.

(4) Mapping rainfall intensity to economic loss

The final step in the model links standardized rainfall intensity to flood-induced losses by applying an empirical damage function derived from Zhang et al. [57]. We simplify their original estimation of economic losses in Guangdong Province by translating it into a proportional loss of community functionality. The relationship is formalized as:

(6) $$L_{\text{ratio},\;t}=0.1069\;\times I_{std,\;t}+0.2903$$

where $L_{\text{ratio},\;t}$ represents the theoretical flood loss ratio, a proportion of total loss at tick *t*; and $I_{std,\;t}$​ represents standardized rainfall intensity at tick *t*. Substituting the maximum intensity value of 5 into Eq. (6) yields a peak theoretical flood loss ratio of 0.8248.

### Loss Sharing Module

This module simulates how the community reaches out to its connected stakeholders for external support to distribute and share flood-induced losses, thus reducing the immediate impacts.

(1) Basic rules

The loss-sharing mechanism operates under the following rules:

• The community node initiates loss-sharing requests sequentially to linked stakeholders. Stakeholders decide whether to respond based on their response probability and resource availability. Stakeholders with less than 1 unit of resources are unable to respond.
• Each time a stakeholder responds successfully, it expends 1 unit of its resources. Resources are periodically replenished based on stakeholders’ type or through the funding measure.
• After responding, a stakeholder remains active for a fixed period and can continue providing support during that window. Successful activations increase inter-stakeholder trust, improving the effectiveness of future collaborations.
• If initial outreach is insufficient, the stakeholder nodes escalate the request to linked stakeholders within a network distance of 1. At each tick, a stakeholder can respond successfully to at most one request.

(2) Calculating loss-sharing proportion

The proportion of flood-induced loss shared among stakeholders is calculated with a sigmoid function S(·) to normalize the values to the range of 0 to 1. This formula is designed to ensure that stronger, more trusted connections and higher community resistance capacity jointly contribute to a greater proportion of reduced loss:

(7) $$L_{\text{share},\;t}=S\left(C_{\text{resist}}\times \sum_{\left(i,j\right)\in C}\;\left({E_{ij}}^{W_{E}}\times T{r_{ij}}^{W_{Tr}}\right)\times \varepsilon \right)$$

where $L_{\text{share},\;t}$ denotes the normalized proportion of total flood-induced loss shared or mitigated through stakeholders’ responses; $C_{\text{resist}}$ is the community’s resistance capacity, reflecting its inherent ability to withstand flood impacts; $E_{ij}$ is the effectiveness of the link from the source node i to the target node j, representing residents’ perceived usefulness of the link in flood response; $W_{E}$ is the weight assigned to link effectiveness; $Tr_{ij}$ is the trust level between node i and node j; $W_{Tr}$ is the weight assigned to trust; and ε is a stochastic factor capturing uncertainty. The summation aggregates support from all active, responding node pairs (i,j)∈C, where C denotes the set of existing connections in the governance network.

Based on expert scoring and insights gathered from resident workshops, we assign weights of $W_{E}$ = 0.9 to link effectiveness and $W_{Tr}$ = 0.1 to trust. This weighting reflects the dominant role of perceived effectiveness in shaping collaborative loss-sharing, while accounting for the contribution of inter-stakeholder trust. Therefore, the equation captures a behavioral pattern in emergency contexts: when seeking immediate loss mitigation, the functional utility of a connection (the resources and assistance it can deliver) matters more than relational history.

(3) Updating community functionality during flood

As the flood progresses and stakeholders continue to help reduce flood impacts, the community functionality is updated iteratively as:

(8) $$F_{t}=F_{t-1}\times \left(1-\;L_{\text{ratio},\;t}\times \left(1\;-\;L_{\text{share},\;t}\right)\right)$$

where $F_{t}$ represents the community functionality at tick t; $F_{t-1}$ is the community functionality at the previous tick; $L_{\text{ratio},\;t}$ represents the theoretical flood loss ratio at tick *t*, generated in Eq. (6); and $L_{\text{share},\;t}$ denotes the normalized proportion of shared or reduced flood loss. To clarify Eq. (8), current community functionality during the flood equals previous functionality minus the portion lost to flood impacts. Only the unshared part of the loss—that is, the proportion not mitigated through loss-sharing cooperation—is applied to reduce functionality.

### Post-flood Recovery Module

*(1) Basic rules*

The post-flood recovery operates under similar rules as the loss-sharing process.

*(2) Calculating recovery contribution*

If a stakeholder responds successfully during the post-flood phase, its effective contribution to restoring the community functionality is calculated using a sigmoid function S(·)to normalize the output. The formula incorporates the helper’s structural position, ensuring that central broker stakeholders, operating through trusted and effective links alongside the community’s inherent capacity, maximize the recovery impact. The proportion of stakeholders’ recovery contribution is calculated as:

(9) $$\begin{array}{c} L_{\text{recover},\;t}=S\left(C_{\text{recover}}\times \sum_{\left(i,j\right)\in C}\left({E_{\mathrm{ij}}}^{W_{E}}\times T{r_{\mathrm{ij}}}^{W_{\mathrm{Tr}}}\times B{C_{i}}^{W_{\mathrm{BC}}}\right)\times \varepsilon \right) \end{array}$$

where $L_{\text{recover},\;t}$ represents the normalized recovery contribution provided to the community; $C_{\text{recover}}$ is the community’s recovery capacity, reflecting the community’s inherent ability to restore its functionality over time;$E_{ij}$ is the effectiveness of the link from node i to node j, representing residents’ perceived usefulness of the link in supporting post-flood recovery; $W_{E}$ is the weight assigned to link effectiveness; $Tr_{ij}$ denotes the trust level between nodes i and j; $W_{Tr}$ is the weight assigned to trust; $BC_{i}$ is the normalized betweenness centrality of the source node i; $W_{BC}$ is the weight assigned to betweenness centrality; and ε is a stochastic factor representing uncertainty. The summation is over all connected node pairs (i,j)∈C, similar to Eq. (7).

Betweenness centrality (BC) quantifies how frequently a node serves as a bridge between other nodes [58]. We include BC in the recovery contribution for two reasons.

First, BC is frequently utilized in disaster research to measure a stakeholder’s brokerage position and its associated information and coordination advantages [[59], [60], [61], [62], [63]]. Nodes with high BC are in a special position, spanning organizational boundaries and connecting otherwise isolated groups [61]. This position allows them to mobilize and distribute resources from heterogeneous stakeholder groups and facilitate efficient flow of information and aid [64]. Empirical evidence shows that betweenness centrality is the most significant variable in building adaptive capacity [65]. By connecting dispersed parts of the local stakeholder network, these actors provide redundancy and alternative resource supplies, thereby enhancing response and recovery activities more effectively [66].

Second, this selection is supported by our field observations and feedback from local residents. The role of BC appears particularly significant during the recovery and reconstruction phases (compared to the immediate, time-constrained emergency response phase). Based on community residents’ perceptions, these high-BC actors (like NGOs) connect the community with external expertise, funding, and organizational support that would otherwise remain inaccessible, such as doctors from local hospitals providing community health consultations and outreach, or funding from private companies for reconstruction and climate adaptation.

Based on expert scoring and discussion in resident workshops, we assign weights of $W_{E}$ = 0.8 to link effectiveness, $W_{Tr}$ = 0.1 to trust, and $W_{BC}$ = 0.1 to betweenness centrality. This weighting emphasizes the critical role of perceived effectiveness in facilitating recovery, while acknowledging that trust and network position (captured by BC) provide additional contributions.

*(3) Updating community functionality post-flood*

During the recovery phase, community functionality is gradually restored as stakeholders contribute support. The post-flood functionality is updated iteratively as:

(10) $$F_{t}=F_{t-1}\times \left(1+\;L_{\text{recover},\;t}\right)$$

where $F_{t}$ is the community functionality at tick *t*; $F_{t-1}$ is the community functionality at the previous tick; $L_{\text{recover},\;t}$ is the normalized proportion of recovery contribution at tick *t*. Once the community functionality value reaches 100, recovery operations cease, as the community system is considered fully restored.

### Flood Resilience Measures Module

This module simulates the implementation of five flood resilience measures: Flood Mitigation Infrastructure, Early Warning System, Community-Based Disaster Education, Emergency Response and Community Support, and Disaster Contingency and Climate Adaptation Funds. Each measure consists of 4 to 10 empirically derived activation pathways, with each pathway represented as a sequence of stakeholder nodes. These pathways were constructed using field data. More details about pathways are provided in the Appendix of Zhu et al. [44]. Each operates within its designated disaster phase (pre-disaster, during-disaster, or post-disaster) to enhance community resistance capacity and recovery capacity through network-based activation processes.

Each measure is executed once during its corresponding disaster phase, with all predefined pathways being simultaneously attempted for activation. The process starts with an initial stakeholder, whose participation is determined by response probability and available resources. If the initiating node is successfully activated, the intervention proceeds through the network according to a structured sequence of stakeholders.

For activation to proceed, each stakeholder must be connected to a previously activated node. If any node along the pathway fails to activate due to insufficient response probability, resource limitations, or lack of network connectivity, the entire pathway is disrupted and activation halts. The measure only takes effect if all nodes within the pathway are activated successfully, and the activation chain reaches the community node.

When activation successfully spans the entire pathway to the community, its resistance capacity and recovery capacity are enhanced by predefined effect sizes, capped at an upper bound of 1. Both capacities are initially set to 0.5 and are adjusted based on the intervention’s impact level. These enhancements apply only if the final node in the activated pathway maintains a direct connection to the community node.

### Trust Update Module

Trust (T) in the governance network is dynamic and evolves through repeated interactions among stakeholders. When a help request is transmitted from a source node to a target node, and the target node is successfully activated (that is, it contributes to loss sharing, post-disaster recovery, or the implementation of flood resilience measures), the trust level between the two nodes will increase. We further adopt the diminishing-returns trust growth mechanism proposed by Sutcliffe and Wang [50], in which each positive interaction contributes to trust formation, but the marginal gain decreases as the existing trust level rises. This formulation departs from linear increase and instead yields a logarithmic trajectory that gradually approaches a stable upper bound.

Following Sutcliffe and Wang [50], the trust update process consists of three steps. First, the compression interval (CI) is used to determine how quickly the trust increment contracts as the trust level approaches its maximum. It is defined as:

(11) $$CI=\frac{MaxCR-MinCR}{MaxTrust}$$

where MaxCR is the maximum trust increase when trust is at its minimum of zero; MinCR is the minimum increment when trust is near its maximum; and MaxTrust represents the upper bound of the trust scale.

Second, the trust increment for the next tick ($\Delta Tr_{t+1}$) is computed as:

(12) $$\Delta Tr_{t+1}=MaxCR-CI\times Tr_{t}$$

which ensures that agents with low trust values receive proportionally larger increases from successful interactions, while agents with high trust experience only marginal gains.

Third, trust at the next tick ($Tr_{t+1}$) is updated according to:

(13) $$Tr_{t+1}=Tr_{t}+\Delta Tr_{t+1}$$

In our model, trust is set from 0 to 1; MinCR is 0 and MaxTrust is 1. Upon integrating (11), (12), (13) and substituting the specified parameter values, the trust level at next tick ($Tr_{t+1}$) update equation simplifies as:

(14) $$Tr_{t+1}=\min\left(1,Tr_{t}+MaxCR\times \left(1-Tr_{t}\right)\right)$$

where MaxCR is the maximum trust increment when trust equals zero; $Tr_{t}$ is the trust level at tick t.

Because direct empirical estimation of trust evolution is difficult, the value of MaxCR requires careful empirical calibration. In this model, the maximum trust increment was set to 0.001 based on two considerations. First, expert scoring and discussions with residents indicated that trust in local governance networks grows slowly and incrementally. This pattern was also emphasized by our NGO partners, who have worked in the study communities for three and seven years, respectively, and have observed first-hand how trust builds gradually through repeated interactions, implying that MaxCR must remain small. To finalize the value, we presented the preliminary expert scoring, model outputs, and sensitivity analysis to community representatives for deliberation and validation.

Second, the model generates a high frequency of interaction due to the density and activity level of the governance network. Therefore, a relatively small MaxCR is necessary to avoid unrealistically rapid inflation of trust values toward the upper bound of 1, while remaining large enough to permit meaningful trust dynamics that influence adaptation and recovery processes over time. Sensitivity analysis on different maximum trust increments is presented in a later section.

### Resource Constraints Module

The module incorporates a dynamic resource allocation process to simulate how stakeholders operate under budget constraints. Each stakeholder is assigned an initial resource budget, which is gradually depleted as they engage in response activities and replenished through periodic inflows. This includes three key processes: resource depletion, periodic replenishment, and flood resilience measures implementation.

(1) Resource depletion in response activities

Model users can adjust initial resources. The baseline configuration initializes each stakeholder with 24 units. Values set too low (e.g., 5) cause premature inactivity, preventing community support. Conversely, high values (e.g., 500) render resource constraints meaningless. This 24-unit baseline aligns with the maximum flood duration of 24 ticks. It allows agents to survive the peak event but ensures scarcity pressures surface during the recovery phase.

Stakeholders expend resources each time they are successfully activated for loss sharing or post-disaster recovery. Each successful response reduces their available resources by 1 unit. Once a stakeholder’s resource level reaches zero, they cannot be activated.

(2) Periodic resource replenishment

Parameters related to resource replenishment can be set by model users. In the current setting, governmental actors receive 10 units of replenishment every 16 ticks. Non-government actors receive 6 units every 8 ticks. Because empirical estimates for these two parameters are inherently uncertain, specific parameter values were determined through a standard Genetic Algorithm (GA) optimization. The GA optimization is well-suited for ABM parameter tuning, which involves a large, nonlinear, and interaction-dependent search space where small parameter changes could substantially alter system behavior. Compared with local search or gradient-based methods, GA can better explore multiple regions of the parameter space simultaneously and avoid converging prematurely to a local solution [67]. Details about the GA optimization are provided in the Appendix.

(3) Flood resilience measures implementation

When a resilience measure is implemented along a planned pathway, each activated node incurs a fixed cost of 10 resource units, regardless of whether the measure succeeds or fails. Successful implementation of the “Disaster Contingency and Climate Adaptation Funds” results in a net replenishment of 20 resource units for all involved stakeholder nodes. These two values were determined through expert scoring to reflect the perceived implementation burden and expected benefits. This structure ensures that resilience measures create both short-term resource pressures and potential long-term benefits, consistent with empirical accounts of community-based adaptation measures.

### Resilience Assessment Module

This study adopts the social resilience framework of Schweitzer et al. [15] to assess community resilience from two dimensions: (1) structural dimension, defined as robustness, which reflects the system property to withstand shocks; and (2) dynamic dimension, defined as adaptivity, which represents the ability to overcome adversity.

(1) Calculating robustness

Robustness measures how well a community can preserve its functionality during a flood event with the assistance of connected stakeholders [68]. Area under the curve (AUC) is used to integrate the community’s functionality over the entire analysis period, from the onset of rainfall to its end. The calculation of Robustness (R) is derived from the cumulative area of three functionality trajectories: 1) Ideal community functionality ($F_{I}$). It is a counterfactual baseline representing an undisturbed community where functionality remains constant at 100 throughout the flood duration; 2) Worst-case community functionality ($F_{W}$). It represents the theoretical functionality without any stakeholder’s assistance. In this trajectory, the community receives no external help; 3) Observed community functionality with stakeholders’ assistance ($F_{A}$). It denotes the observed community functionality with stakeholders’ aid in sharing flood-induced loss and implementing flood resilience measures. Robustness (R) is then calculated as the ratio of saved community functionality to the maximum potential functionality loss:

(15) $$R=\frac{AUC\left(F_{A}\right)-AUC\left(F_{W}\right)}{AUC\left(F_{I}\right)-AUC\left(F_{W}\right)}$$

The denominator, $AUC\left(F_{I}\right)-AUC\left(F_{W}\right)$, represents the cumulative potential loss the community would face under the worst-case scenario. The numerator, $AUC\left(F_{A}\right)-AUC\left(F_{W}\right)$, quantifies the portion of that potential loss successfully saved through stakeholder interventions. Therefore, R is a standardized score, the proportion of potential flood-induced losses mitigated by the governance network. A higher R value indicates that stakeholder actions were more effective in mitigating flood impacts and sustaining community functionality.

(2) Calculating adaptivity

Adaptivity captures the system’s dynamic capacity to recover in the aftermath of a disturbance and adapt to changing environments. In the model, adaptivity is measured using two complementary indicators. We first measure recovery rapidity to capture how quickly the community recovers [69]. Yet, adaptivity requires continuous collective effort and internal responses rather than just restoration [70]. Therefore, we also measure governance network activity, designed to reflect the level of internal responsiveness and the overall flexibility and active engagement within the system. A highly active network possesses more available options and the flexibility to reconfigure itself, aligning with the facet of adaptivity as the “potentiality to attain different states” [15].

Recovery rapidity ($A_{T}$) measures how quickly the community restores its functionality after flood-induced disruptions. It is defined as:

(16) $$A_{T}=\frac{T_{f}}{T_{r}}$$

where $T_{f}$ is the duration of the rainfall; $T_{r}$ is the total time required to restore community functionality to 100 from the onset of the rainfall, which means it includes the flood duration itself. A value of $A_{T}$ approaching 1 indicates a rapid recovery, whereas a value approaching 0 reflects a slow or prolonged recovery process.

Governance network activity ($A_{N}$) is the average engagement level of stakeholders embedded in the governance network across three disaster phases and is calculated as:

(17) $$A_{N}=\frac{1}{3}\times \left(\frac{N_{1}}{N_{0}\times \left(t_{f}-t_{0}\right)}+\frac{N_{2}}{N_{0}\times \left(t_{r}-t_{f}\right)}+\frac{N_{3}}{N_{\text{max}}}\right)$$

where $N_{0}$ is the total number of nodes (stakeholders) in the governance network; $t_{0}$ represents flood start time; $t_{f}$ represents flood end time; $t_{r}$ denotes recovery completion time; $N_{1}$ represents the number of stakeholder responses during the flood loss-sharing phase (from $t_{0}$ to $t_{f}$); $N_{2}$ denotes the number of stakeholder responses during the recovery phase (from $t_{f}$ to $t_{r}$); $N_{3}$ is the total number of responses related to the implementation of flood resilience measures; and $N_{\text{max}}$ denotes the theoretical maximum responses if all nodes along each pathway, which refers to the multi-stakeholder sequence through which a measure is transmitted and coordinated, are activated.

Each term in the parentheses represents a standardized ratio between the actual and theoretical maximum possible responses in each phase. For instance, the denominator $N_{0}\times \left(t_{f}-t_{0}\right)$ represents the maximum number of potential responses if all stakeholders were active at every time step during the flood event. A higher $A_{N}$ value reflects a more flexible, active governance network, indicating more adaptive options available in the system.

Overall adaptivity (A) combines these two metrics as:

(18) $$A=\alpha A_{T}+\left(1-\alpha \right)A_{N}$$

The weight α = 0.6 was determined based on expert scoring and community workshops. This weighting emphasizes the critical importance of rapid recovery while still accounting for governance-level engagement.

*(3) Calculating community resilience*

Community resilience to floods refers to the capacity of a community to withstand and recover from disaster impacts [15]. Community resilience (Ω) is conceptualized as a function of robustness (R) and adaptivity (A).

(19) Ω=βR+(1−β)A

where the weighting parameter β is set to 0.5. This equal weighting reflects the theoretical premise that robustness and adaptivity, representing the structural and dynamic dimensions, are equally important to community resilience [15,70]. This parameterization is further supported by expert scoring and stakeholder validation.

Community resilience is measured upon the completion of the full response and recovery cycle. While this model quantitatively measures short-term resilience via robustness and adaptivity, the simulated outcome extends beyond merely reducing impacts or fostering recovery; the community itself is fundamentally different from its pre-disaster state [71]. The model’s dynamic processes capture the emergence of long-term adaptation trajectories and structural transformations, particularly through increased trust and community capacity. The level of trust between stakeholders is enhanced by successful collaborations. Successfully implemented physical and non-physical measures lead to improvement in community resistance capacity and recovery capacity. They become the new baseline for future interactions and represent a structural transformation in the governance network and a persistent improvement in community capacities, thus preparing better for future changes.

## Method validation

The dynamic nature and parametric complexity of social systems often make the precise reproduction of empirical data infeasible [72]. For ABMs grounded in detailed field evidence, validity can be assessed by how accurately the model represents stakeholders’ views [73]. Presenting models to stakeholders and using their feedback for refinement is widely used in ABM research [43,74]. Since our model aims to understand social mechanisms rather than predict specific flood losses, we prioritize stakeholder validation. This approach focuses on verifying underlying processes to reproduce key system patterns, rather than fitting the community functionality curve to scarce observed data. We therefore calibrated and validated the ABM using multiple approaches, with stakeholder feedback as the primary benchmark to ensure that the model accurately represents the patterns of flood governance dynamics.

Firstly, empirical grounding primarily relies on fieldwork and household survey data, which informed baseline trust levels, help-seeking behaviors, and stakeholder roles. The model and its key parameters were further presented, discussed and refined, through participatory resident workshops and expert scoring, ensuring that modeled social dynamics, such as the “near-to-far” assistance activation logic, faithfully reflect observed community behaviors. Stakeholders further validated the plausibility of the model’s assumptions, mechanisms and outcomes.

Secondly, sensitivity analysis systematically examined key parameters related to floods, trust, resources, and analytical weights, confirming model stability and interpretable responses across scenarios. Convergence tests established a minimum of 200 simulation runs per scenario to account for output variability. Details on the determination of the minimum simulation runs and sensitivity analysis are provided below.

Thirdly, we strengthened validation of both model processes and outcomes. We first drew on field observations and contemporary news reports documenting actual flood responses, such as the coordinated emergency actions during the June 2022 urban flooding event and Super Typhoon Hato (2017) and Super Typhoon Mangkhut (2018). These records support the model’s representation of the sequential activation of governance actors, from subdistrict offices and volunteer teams to social workers. We then validated recovery time against external quantitative estimates. A recent city-scale study using a parametric recovery-curve framework found that 59.1% of 220+ Chinese cities, including Guangzhou, were able to return to pre-disaster performance levels within 4.9–9.1 days [75]. Our baseline simulations produce a similar recovery time of 185.55 hours (around 7.7 days) across 200 runs.

### Determining minimum simulation runs

**Fig. 3:**
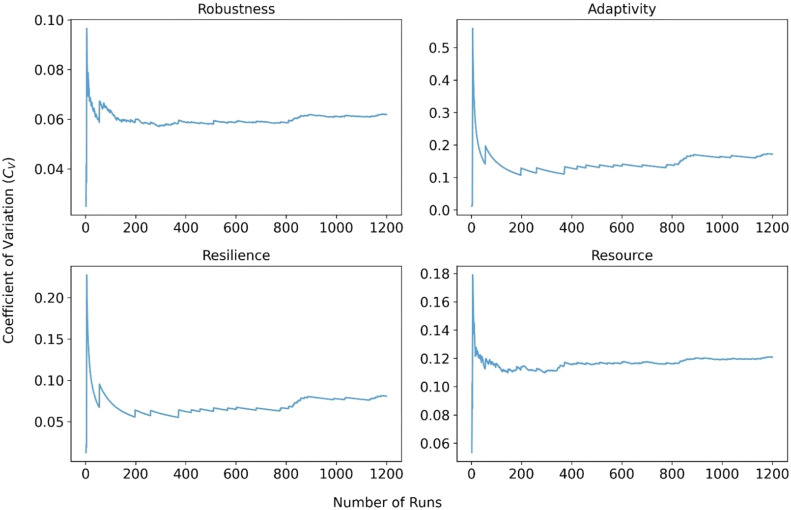
Estimating minimum simulation runs based on output variability (cᵥ) across robustness, adaptivity, resilience, and resource. Simulations were run under a controlled baseline scenario: the model simulated the 100-year rainfall event, initial trust levels were uniformly set to 0.5, all flood resilience measures were deactivated, and all other trust increment and resource parameters were held at their baseline values.

In agent-based modeling, particularly when simulating stochastic systems such as governance networks, determining the minimum number of simulation runs is critical for balancing output reliability and computational efficiency. We evaluated how output variability, measured by the coefficient of variation (*C_V_*), responds to increasing numbers of replications [76,77]. *C_V_* was computed as the standard deviation divided by the mean value. As shown in Fig. 3, our analysis reveals that beyond approximately 200 runs, the relative variability of outputs stabilizes, suggesting that additional replications yield minimal gains in results precision (see Fig. 3). Based on this convergence pattern, we set 200 as the minimum number of simulation runs required to ensure output stability.

### Sensitivity analysis

To evaluate the robustness of the model under parameter uncertainty, a one-at-a-time (OAT) sensitivity analysis was conducted. OAT involves varying one input parameter across a predefined range while holding all other parameters constant at their baseline values [76]. This approach was chosen because it is well-suited as a starting point for sensitivity analysis in ABMs, especially when the aim is to investigate underlying mechanisms and emergent patterns rather than average effects across the full parameter space [77]. OAT is also advantageous for identifying nonlinearities and tipping points, which are often masked in global variance decomposition techniques [77,78].

The parameters included in the analysis were selected based on their relevance to the model structure and the absence of detailed empirical data specific to the case study area. Detailed definitions, units, and tested ranges are presented in Table 4. Following the OAT methodology, each parameter was varied across three levels (high, medium, and low) while all other parameters were fixed at their baseline values. The results are visualized using box plots to show the impact of each parameter on the model’s primary outcomes: robustness, adaptivity, and resilience. Each box plot summarizes the median (central line), interquartile range (box), and overall dispersion (whiskers) of the outcome across 200 runs, thereby revealing both systematic trends and changes in output variability.

**Table 4:** Model parameters used in sensitivity analysis.

Model parameters	Description	Unit	Recommended value (baseline)	High scenario	Medium scenario	Low scenario
Flood intensity (return period)	Classification of the rainfall event’s magnitude based on its return period	none	“100-year”	“100-year”	“50-year”	“10-year”
Flood duration	Duration of rainfall-induced flood event	ticks	24	24	14	4
Maximum trust increment	Maximum trust increase when the trust level is at its minimum of 0	none	0.001	0.0015	0.001	0.0005
Initial resources	Initial resources allocated to all stakeholders	resource units	24	36	24	12
Government actors: resource increment	Resource quantity provided at each replenishment time for government actors	resource units	10	14	10	6
Government actors: replenishment interval	The time between two consecutive replenishments for government actors	ticks	16	20	16	12
Non-government actors: resource increment	Resource quantity provided at each replenishment time for non-government actors	resource units	6	10	6	2
Non-government actors: replenishment interval	The time between two consecutive replenishments for non-government actors	ticks	8	12	8	4
Flood measure cost	The fixed cost incurred by a stakeholder for participating in a flood resilience measure	resource units	10	15	10	5
Flood measure gain	The net resource benefit received by a stakeholder upon the successful implementation of the Disaster Contingency and Climate Adaptation Funds measure	resource units	20	25	20	15
Loss-sharing formula weights (Eq. 7)	Weights assigned to link effectiveness and trust	none	link effectiveness: 0.9, trust: 0.1	0.9, 0.1	0.8, 0.2	0.7, 0.3
Recovery formula weights (Eq. 9)	Weights assigned to link effectiveness, trust and BC	none	link effectiveness: 0.8, trust: 0.1, BC: 0.1	0.9, 0.05, 0.05	0.8, 0.1, 0.1	0.7, 0.15, 0.15
Adaptivity formula weights (Eq. 18)	Weights assigned to Recovery rapidity and Governance network activity	none	Recovery rapidity: 0.6, Governance network activity: 0.4	0.6, 0.4	0.5, 0.5	0.4, 0.6
Resilience formula weights (Eq. 19)	Weights assigned to Robustness and Adaptivity	none	Robustness: 0.5, Adaptivity: 0.5	0.6, 0.4	0.5, 0.5	0.4, 0.6

Note: All simulations were conducted under a controlled baseline scenario. The model was initialized with a 100-year rainfall event, and all agents’ initial trust levels were uniformly set to 0.5. All flood resilience measures were activated for evaluating the effects of resource-related parameters. To account for the stochasticity inherent in agent-based modeling, each configuration was replicated 200 times.

**Fig. 4:**
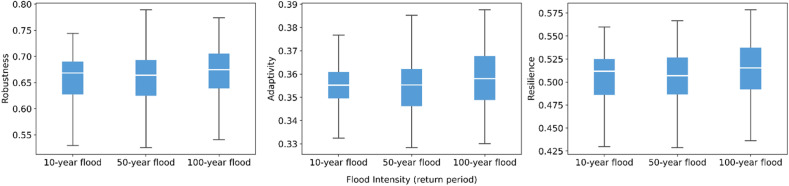
Sensitivity of robustness, adaptivity, and resilience to flood intensity.

As shown in Fig. 4, flood intensity has a relatively limited effect on model outcomes. The median values of robustness, adaptivity, and resilience shift only slightly across return periods, and the interquartile ranges remain largely overlapping. This suggests that the magnitude of the external shock has a muted effect on system performance when the duration of the event is held constant. This stability stems from the model’s emphasis on inter-agent collaboration, which functions as a buffering mechanism that offsets the salience of shock intensity.

**Fig. 5:**
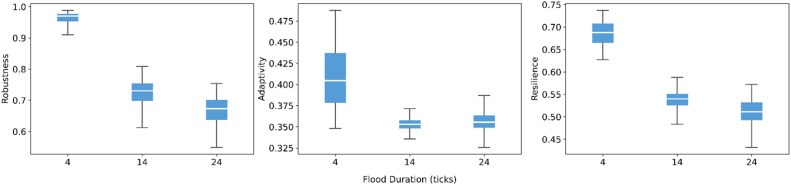
Sensitivity of robustness, adaptivity, and resilience to flood duration.

Flood duration (Fig. 5), by contrast, exerts a much more pronounced effect. As the duration increases from 4 to 24 ticks, both robustness and resilience decline because longer events generate larger cumulative flood losses. Adaptivity is highest for short events and stabilizes at a lower level for medium and long duration. The sharp drop in robustness is particularly notable, indicating that prolonged exposure can overwhelm the absorptive capacity of the governance system. This shows the structural limits of local coping capacities under persistent stress.

**Fig. 6:**
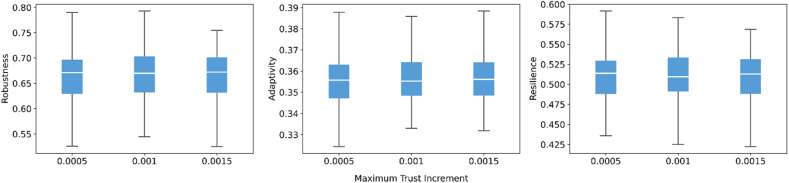
Sensitivity of robustness, adaptivity, and resilience to maximum trust increment.

The maximum trust increment parameter has a limited influence on model outputs in Fig. 6. As the increase rate rises, all three indicators remain almost entirely stable. Across the tested range, the medians and spread of robustness, adaptivity, and resilience remain almost unchanged. This pattern is consistent with the model’s trust update rule: the maximum increment applies only when trust is near zero, and marginal gains diminish as trust level increases. As a result, moderate adjustments to the maximum increment do not translate into large differences in resilience outcomes.

**Fig. 7:**
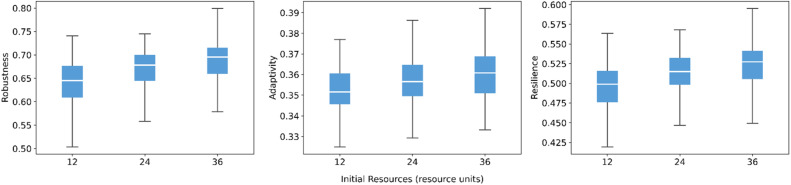
Sensitivity of robustness, adaptivity, and resilience to initial resources.

Initial community resources have a clear and positive effect (Fig. 7). Increasing initial resources from low to high leads to a rise in the median values of all three indicators, with robustness and resilience improving most visibly. This result aligns with the high-resource demand scenario in the sensitivity analysis (where all resilience measures are activated), suggesting that greater initial capacity enables the governance network to sustain more extensive loss-sharing and recovery actions.

**Fig. 8:**
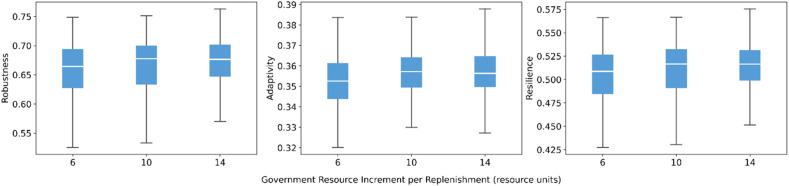
Sensitivity of robustness, adaptivity, and resilience to resource increment of government actors.

**Fig. 9:**
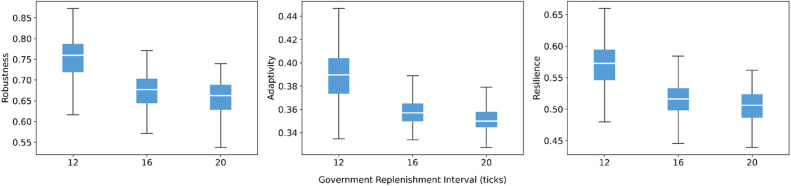
Sensitivity of robustness, adaptivity, and resilience to replenishment interval of government actors.

Government resource increment per replenishment shows a mild but consistent positive effect (Fig. 8). Higher increments are associated with small increases in robustness, adaptivity, and resilience, while the spread of results remains stable. The interval of government replenishment is more influential (Fig. 9). Lengthening the replenishment interval systematically reduces the medians of all three indicators and slightly widens the outcome distributions. This indicates that less frequent provision of governmental resources undermines system performance. These results underscore that, in high-resource-demand situations where all resilience measures are activated, the continuity of governmental support matters more than marginal changes in the size of each replenishment.

**Fig. 10:**
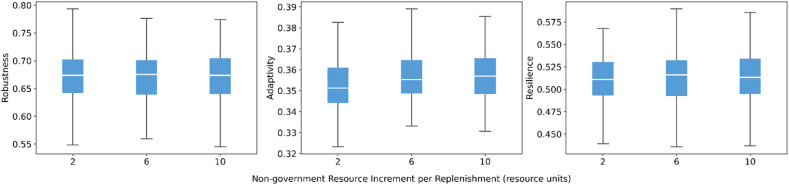
Sensitivity of robustness, adaptivity, and resilience to resource increment of non-government actors.

**Fig. 11:**
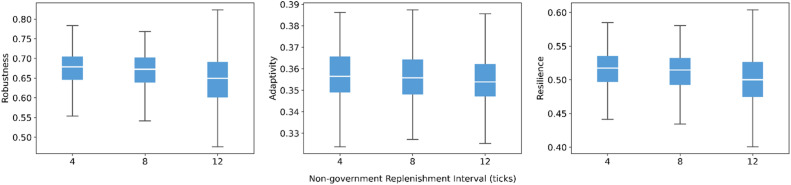
Sensitivity of robustness, adaptivity, and resilience to replenishment interval of non-government actors.

For non-government actors, similar patterns emerge. Increasing the resource increment per replenishment produces gentle improvements in adaptivity and resilience, with almost unchanged robustness (Fig. 10). Extending the replenishment interval from short to long periods leads to slight declines in all three indicators (Fig. 11), but the differences in medians are small and the boxplots remain strongly overlapping. The smaller, more frequent replenishments received by non-government actors provide more continuous support. This continuity renders reductions in replenishment frequency (i.e., longer intervals) less impactful. As a result, these scheduling changes only modify capacity at the margin and do not substantially alter system-level resilience.

**Fig. 12:**
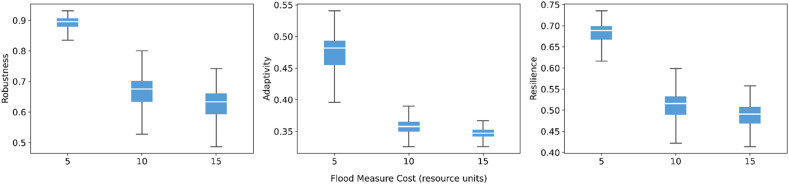
Sensitivity of robustness, adaptivity, and resilience to flood measure cost.

**Fig. 13:**
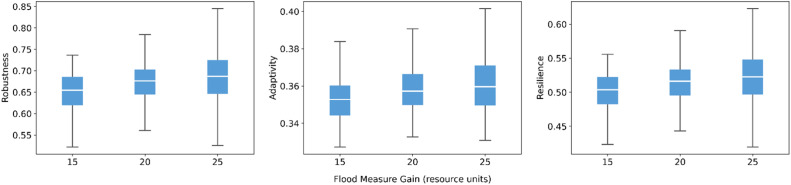
Sensitivity of robustness, adaptivity, and resilience to flood measure gain.

The cost and gain of implementing flood resilience measures behave as expected. Increasing implementation costs leads to lower robustness, adaptivity, and overall resilience (Fig. 12). The decline is much steeper when the cost increases from 5 to 10, while the drop from 10 to 15 is noticeably smaller. This suggests that initial cost increases place the system under sharp resource strain, whereas additional cost burdens lead to diminishing marginal losses. Conversely, increasing the net gain from successful implementing “Disaster Contingency and Climate Adaptation Funds” measure leads to gradual improvements in all three metrics (Fig. 13). The upward shift in medians is moderate but consistent, showing that additional rewards for successful measures translate into more resources circulating in the governance network and, in turn, stronger resilience performance.

**Fig. 14:**
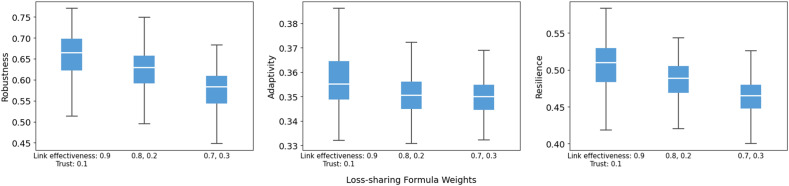
Sensitivity of robustness, adaptivity, and resilience to loss-sharing formula weights.

**Fig. 15:**
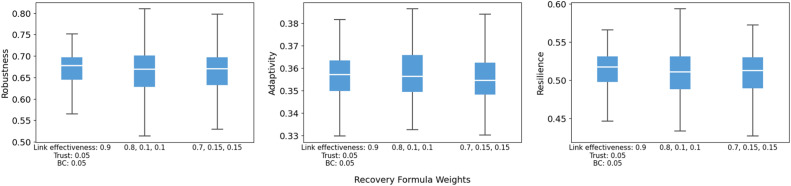
Sensitivity of robustness, adaptivity, and resilience to recovery formula weights.

For the loss-sharing and recovery formulas, the model shows limited sensitivity to moderate re-weighting of inputs, and the direction matches our expectations. In the loss-sharing formula (Fig. 14), shifting weight from link effectiveness toward trust slightly lowers robustness and resilience, while adaptivity changes little. As expected, placing more weight on link effectiveness increases resilience because it is the key driver of preserving core functionality and accelerating early recovery. By contrast, the recovery formula weights (Fig. 15) have little influence, with largely overlapping distributions and near-constant medians across robustness, adaptivity, and resilience.

**Fig. 16:**
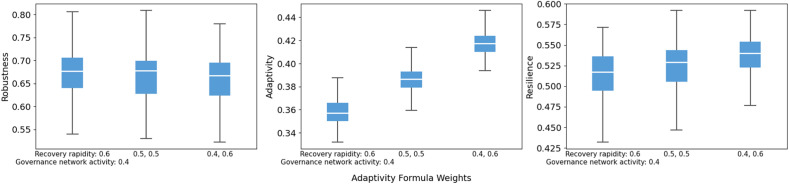
Sensitivity of robustness, adaptivity, and resilience to adaptivity formula weights.

**Fig. 17:**
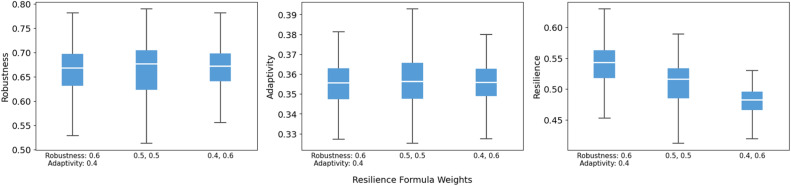
Sensitivity of robustness, adaptivity, and resilience to resilience formula weights.

For the adaptivity and resilience formula weights, shifts follow the expected direction. In the adaptivity formula, increasing the weight on governance network activity raises adaptivity and slightly increases resilience, whereas placing more weight on recovery rapidity lowers adaptivity (Fig. 16). In the resilience formula, shifting weight toward adaptivity reduces the resilience median, while assigning more weight to robustness increases it (Fig. 17). This is because robustness values are generally higher than adaptivity in the simulations. Overall, reweighting moves the composite indices in predictable ways without drastic fluctuations.

Taken together, the sensitivity results demonstrate that the model’s resilience outcomes are structurally stable under moderate, empirically plausible parameter variations. Flood duration is the most influential hazard-side factor, as the system buffers short-term shocks but struggles to sustain performance under prolonged stress. On the governance side, resource availability and continuity on the network matter more than marginal changes in many behavioral parameters. These experiments show that resilience is an emergent property of boundedly rational behavior and localized interactions, where alternative rule configurations could shift the system onto divergent resilient pathways.

## Brief model user’s guide

### How to use the model

1. Importing data

To begin, users must import two CSV files: one providing the network structure and another about nodes’ attributes. The network data file should contain at least four columns: source-node, target-node, link-effectiveness, and link-type. The node attribute file must include response-probability, response-duration, and node-type. Upon import, stakeholder nodes are displayed in grey, community nodes in blue, and stakeholders who successfully respond will change to yellow to indicate activation.

Users need to customize specific governance pathways by editing the to-flood-resilience-measure procedure in the NetLogo code. As guided by the inline comments, users need to replace the stakeholder node names within the brackets. This enables custom assignment of which actors are responsible for which flood resilience measures.

2. Setting simulation parameters

Users can adjust key parameters directly on the interface:

• Flood intensity, with three return period options (10, 50, 100-year flood).
• Flood duration, which ranges from 4 to 24 ticks (hours).
• Maximum trust increment and initial trust levels toward both government and non-government actors, adjustable between 0 and 1.
• Resource replenishment settings, including initial resources, government actors’ resource increment and replenishment interval, non-government actors’ resource increment and replenishment interval, and the cost and gain of flood measures.
• Five flood resilience measures, which can be turned on or off: (1) Flood Mitigation Infrastructure, (2) Early Warning System, (3) Community-Based Disaster Education, (4) Emergency Response and Community Support, and (5) Disaster Contingency and Climate Adaptation Funds.

3. Calibrating parameters

Users must calibrate specific parameters in the model to match local contexts. We provide a Calibration Checklist (Table 5) to guide users through the necessary parameter adjustments. The determination and verification of these parameter values should involve consultation with local residents and other key stakeholders represented in the model to ensure the settings accurately reflect local realities.

**Table 5:** Calibration checklist.

Calibration module	Parameter/formula	Recommended validation source
Environment	Rainfall intensity equation	Meteorological bureau, official IDF/design storm report
	Flood damage curve	Historical disaster records, literature review
Trust	Maximum trust increment	Participatory workshops, stakeholder interviews and surveys
Resource	Initial resources	Official documents and records, participatory workshops, expert scoring, fieldwork, stakeholder surveys, GA optimization
	Government actors: resource increment	
	Government actors: replenishment interval	
	Non-government actors: resource increment	
	Non-government actors: replenishment interval	
	Flood measure cost	
	Flood measure gain	
Analytical weights	Loss-sharing formula weights	Literature review, participatory workshops, expert-scoring
	Recovery formula weights	
	Adaptivity formula weights	
	Resilience formula weights	

4. Running simulations and batch experiments

After configuring the settings, clicking the “setup” button initializes the model by generating the governance network based on the imported data. Clicking the “go” button starts the simulation, where agents respond dynamically to flood events.

To run simulations across multiple governance networks (e.g., 40 different network structures), users can use BehaviorSpace. Within the experiment setup, specifying [“network-id” [0 1 39]] instructs the model to iterate through predefined network scenarios automatically. A detailed code example is provided in our open code file to demonstrate how to input and manage multiple governance network structures.

5. Analyzing outputs

Simulation outputs, such as community functionality over time, robustness, adaptivity, overall resilience, recovery time, and resource usage, are exported in CSV format. These can be analyzed using R, Python, or other statistical tools for further comparison and visualization.

6. Practical applications

In our research, community residents have demonstrated strong interest in using the model as a tool for climate change education and collaborative planning. Practitioners, such as local officials, NGO staff, and community organizers, can engage stakeholders in jointly constructing governance networks based on local institutional contexts, simulate system responses under diverse flood scenarios, and interpret the model outputs to inform the co-design of feasible interventions. For instance, users can input governance networks with varying trust levels and flood resilience measures into the ABM. This allows them to identify strategies that enhance community resilience most effectively with acceptable response burdens. These exercises serve as entry points for further discussion on the real-world constraints and opportunities for implementation.

### Things to notice

  (1) This model was developed based on empirical research conducted in urban communities in Guangzhou, China. Users need to recalibrate certain context-specific parameters when applying the model to other settings, as shown in Table 5. Users should note that the primary purpose of the model is to explore how different governance network attributes influence community resilience, rather than to predict precise flood damage.
  (2) Due to data protection reasons, the original governance network data cannot be shared. However, if users require illustrative input samples to test and run the model, the authors are happy to help upon request.
  (3) The model was developed and tested using NetLogo version 6.4. Sensitivity analysis and determining minimum simulation runs were conducted via PyNetLogo [79].

## Limitations

This study has several limitations. First, the validation of the ABM is constrained by the absence of longitudinal and time-series data regarding how stakeholders respond to real flood events over time, as well as the lack of community loss and recovery records. Future efforts to improve model validation could incorporate administrative records, such as emergency dispatch logs, community-level incident reports, or digital help request platforms, to reconstruct response sequences and stakeholder dynamics during disasters.

Second, the model does not account for power asymmetries, conflicting stakeholder priorities, trust erosion, or institutional failure. In some instances, connections within the network may exert negative influences on community resilience, and unmet expectations during disasters can lead to trust decay. Future studies should consider how power, interests, and broader sociocultural factors influence governance dynamics in both positive and negative ways. For those interested in the long-term evolution of resilience, it is important to explore how governance networks and system resilience co-evolve over time. This includes understanding how micro-level interactions and macro-level factors jointly shape how actors enter and position themselves in the governance field and what resources they can mobilize. This perspective can provide insights into the structural transformation of governance networks and their capacity to sustain resilience over time.

Third, the model was developed within the socio-political context of China, where governance is predominantly top-down and centralized. While this context enables clear delineation of authority and coordination pathways, it also limits the generalizability of findings to flood-prone regions with different institutional arrangements. Although the model is adaptable, transferring it to different geographical and institutional settings will require careful calibration and additional validation (see Brief model user’s guide).

## Ethics statements

We confirm that the relevant informed consent was obtained from respondents.

## CRediT authorship contribution statement

**Anqi Zhu:** Conceptualization, Methodology, Software, Formal analysis, Writing – original draft, Visualization, Investigation. **Wenhan Feng:** Conceptualization, Methodology, Software, Validation, Writing – review & editing. **Huan Zheng:** Conceptualization, Validation, Investigation, Writing – review & editing. **Yingxin Huang:** Methodology, Validation, Investigation, Writing – review & editing. **Xin He:** Validation, Investigation, Resources. **Chengying Zhou:** Validation, Investigation. **Liang Emlyn Yang:** Conceptualization, Methodology, Writing – review & editing, Supervision.

## Declaration of competing interest

The authors declare that they have no known competing financial interests or personal relationships that could have appeared to influence the work reported in this paper.

## Data Availability

Data will be made available on request.

## Appendix Supplementary materials

Image, application 1
