# Supplementary material for: An Agent-Based Model for Simulating Flood Governance and Community Resilience
Source: MethodsX. 2026 Feb 13;16:103820. doi: 10.1016/j.mex.2026.103820 (PMC12963990; doi:10.1016/j.mex.2026.103820)
Supplement: Supplementary file 1 [file mmc1.docx]

**Supplementary Materials for：**

An Agent-Based Model for Simulating Flood Governance and Community Resilience

***Genetic Algorithm Optimization for Resource Replenishment Parameters***

We calibrated resource replenishment parameters using a standard Genetic Algorithm (GA) over 50 iterations. To isolate the effect of resource dynamics, the GA optimization was run under controlled conditions. The objective function was to maximize the mean community resilience index. Intensity was set as a 100-year rainfall event, initial trust levels were set at 0.5, and all flood resilience measures were deactivated except for the Disaster Contingency and Climate Adaptation Funds measure. This is to ensure that flood measure cost and gain play a meaningful role in the optimization process. The GA optimization explored a broad yet empirically informed parameter space. The selected ranges were grounded in field observations: government agencies typically receive larger but less frequent budget allocations, while NGOs and community organizations operate under smaller but more frequent funding cycles. These real-world patterns informed the upper and lower bounds for both replenishment intervals and amount during the optimization.
